# FN1 overexpression is correlated with unfavorable prognosis and immune infiltrates in breast cancer

**DOI:** 10.3389/fgene.2022.913659

**Published:** 2022-08-12

**Authors:** Xiu-Xia Zhang, Jun-Hua Luo, Li-Qiang Wu

**Affiliations:** ^1^ Department of Thyroid and Breast Surgery, Linping Campus, The Second Affiliated Hospital of Zhejiang University School of Medicine, Hangzhou, Zhejiang, China; ^2^ Department of Hematology, Zhejiang Provincial Hospital of Chinese Medicine, Hangzhou, Zhejiang, China

**Keywords:** FN1 expression, biomarker, prognosis, immune microenvironment, breast cancer

## Abstract

**Objective:** To investigate the correlation of fibronectin 1 (FN1) expression with prognosis and tumor-infiltrating immune cells in breast cancer (BRCA).

**Methods:** FN1 mRNA and protein expressions were analyzed through Tumor Immune Estimation Resource (TIMER), Gene Set Cancer Analysis (GSCA), Human Protein Atlas (HPA) databases, and immunohistochemical analysis. The clinicopathological characteristics and genetic factors affecting the FN1 mRNA expression were assessed by various public databases. Then, we analyzed the prognostic value of FN1 in BRCA by Kaplan-Meier plotter, receiver operating characteristic, and Cox regression analyses. Further, the UCSC Xena database was used to retrieve TCGA-BRCA expression profiles for functional enrichment analysis and immune cell infiltration analysis. The potential drugs for the BRCA patients with high- FN1 expression were identified using the connectivity map analysis.

**Results:** FN1 was upregulated in BRCA tissues compared with normal tissues. High FN1 mRNA expression was correlated with poor clinical outcomes and had good performance in predicting the survival status of BRCA patients. Further, Cox regression analysis showed that FN1 was an independent prognostic factor for predicting the overall survival of patients with BRCA. Moreover, hypermethylation of FN1 contributed to a better prognosis for BRCA patients. Functional enrichment analyses revealed the ECM-receptor interaction pathway and focal adhesion as the common pathways. Moreover, FN1 showed a significant association with tumor-infiltrating immune cells and immune checkpoint inhibitors. Several drugs such as telmisartan, malotilate, and seocalcitol may have therapeutic effects in BRCA patients with high FN1 expression.

**Conclusion:** FN1 might serve as a novel prognostic biomarker and a novel therapeutic target for BRCA. Besides, the association of FN1 with immune cells and immune checkpoint inhibitors may provide assistance for BRCA treatment.

## 1 Introduction

Breast cancer (BRCA) is one of the most common malignancies in females worldwide and is responsible for almost 25% of cancer-related deaths in women (Bray et al., 2018). There were approximately 2.3 million women diagnosed with BRCA and 685,000 deaths globally in 2020 (Lei et al., 2021). In the United States, there were 246,660 new cases and 40,450 mortalities from BRCA in 2016 (Siegel et al., 2016), while up to 276,480 women developed BRCA, accounting for 30% of female cancers in 2020 (Siegel et al., 2020). In China, morbidity, and mortality increase year by year as well (Zhang et al., 2021). BRCA is a heterogeneous disease, which can be categorized into subtypes of luminal, human epidermal growth factor receptor 2+ (HER2+), and triple-negative (TNBC) according to the expression of estrogen receptor (ER), progesterone receptor (PR), and HER2 (Yang et al., 2021). Mammography and magnetic resonance imaging are implemented in screening BRCA (Drukteinis et al., 2013). At present, surgery, chemotherapy, radiotherapy, and hormone therapy are available approaches for BRCA treatment (Liu and Ye, 2017). Despite rapid improvement in target therapy methods, the prognosis is still unsatisfactory due to drug resistance and recurrence (Chen et al., 2021). Immunotherapy has emerged as a revolutionary strategy in various tumors, but whether this method would represent a role in BRCA treatment is an open question (de la Cruz-Merino et al., 2019). Therefore, it is of urgency to understand the underlying mechanisms of BRCA and develop potent biomarkers for improving the prognosis of BRCA patients.

The fibronectin (FN) family is widely expressed by multiple cell types and participates in cell adhesion and migration processes during host defense, blood coagulation, wound healing, and embryogenesis, as well as in cell proliferation (Pankov and Yamada, 2002; Gao et al., 2016). As a member of the FN family, FN1 encodes a glycoprotein that is expressed in plasma as a soluble dimer and on the surface and extracellular matrix (ECM) as a dimer or polymer (Geng et al., 2021). Besides, FN1 is involved in NKp46 receptor-mediated interferon-γ production by natural killer cells, with respect to the control of tumor architecture and metastasis (Glasner et al., 2018). Previous studies have asserted its involvement in the development of thyroid cancer (Sponziello et al., 2016), renal cancer (Waalkes et al., 2010), and nasopharyngeal carcinoma (Ma et al., 2014). LINC02381 exerts carcinogenic effects in BRCA by the miR-1271-5p/FN1 axis to activate PI3K/AKT pathway (Huang et al., 2022). However, there were rare systemic researches about the function and underlying mechanism of FN1 in BRCA.

Herein, we comprehensively evaluated the relationship between FN1 expression and the prognosis of BRCA patients. After elucidating the biological function and potential regulatory pathways of FN1, we analyzed the association of FN1 mRNA expression with tumor-infiltrating immune cells. This study revealed that FN1 was a potential prognostic and immune-related biomarker in BRCA, which might be a therapeutic target for the BRCA.

## 2 Materials and methods

### 2.1 FN1 expression analysis

Tumor Immune Estimation Resource (TIMER) (https://cistrome.shinyapps.io/timer/) is a web server for comprehensive analysis of tumor-infiltrating immune cells. Firstly, we used TIMER to analyze the FN1 mRNA expression levels in various kinds of tumors. Next, Gene Set Cancer Analysis (GSCA) database (http://bioinfo.life.hust.edu.cn/GSCA/#/), an integrated database for genomic and immunogenomic gene set cancer analysis, was adopted to evaluate the FN1 mRNA expression in BRCA and normal tissues. In addition, we downloaded the data on FN1 mRNA expression and corresponding clinical characteristics from the cBioportal database (https://www.cbioportal.org/) by searching “BRCA” and choosing the “Breast Invasive Carcinoma (TCGA, Firehose Legacy) dataset. Sankey diagram was built based on the “ggalluval” package, which integrated the FN1 RNA-seq data and relevant clinical information. Following this, The Human Protein Atlas (HPA) (https://www.proteinatlas.org/) database was employed to assess the FN1 protein expression in BRCA and normal breast tissues. We searched “FN1” and chose “PATHOLOGY” to obtain representative immunohistochemical images about FN1 protein expression. For validation, immunohistochemistry was conducted to analyze the FN1 protein level in BRCA and normal breast tissues according to the manufacturer’s protocols.

Subsequently, we explored the relationship between FN1 mRNA expression and clinicopathological parameters of BRCA by using Breast Cancer Gene-Expression Miner v4.8 (bc-GenExMiner v4.8) (http://bcgenex.ico.unicancer.fr/BC-GEM/GEM-Accueil.php?js=1). This database is a statistical mining tool of published annotated breast cancer transcriptomic data including DNA microarrays and RNA-seq. It offers the possibility to perform statistical analyses of gene correlation, expression, and prognosis.

Moreover, we selected the Breast Invasive Carcinoma (TCGA, Firehose Legacy) dataset in the cBioportal database to query the genetic alteration frequency and the mutation locations of the FN1 gene in BRCA. Then, the associations of FN1 expression with copy number variation (CNV) and methylation were examined by the Spearman correlation test through the GSCA database. *p* < 0.05 or false discover rate (FDR) < 0.05 was considered to be statistically significant.

### 2.2 Survival analysis of FN1 expression

Survival analysis was performed to examine the prognostic value of the FN1 gene in BRCA patients. The TCGA BRCA data were acquired from the cBioportal database, which was visualized with gene distribution and Kaplan-Meier curves. Patients with complete survival and expression information were enrolled in the study. The receiver operating characteristic (ROC) curves were drawn to evaluate the predictive value of the FN1 gene in distinguishing the survival status of BRCA patients. Besides, the GSE7390 dataset was obtained from the Gene Expression Omnibus (GEO) database (https://www.ncbi.nlm.nih.gov/geo/) to validate the prognostic and predictive value of FN1.

Using the Kaplan-Meier plotter website (http://kmplot.com/), we analyzed the effect of FN1 expression on the OS and DFS of BRCA patients with restricted clinicopathological characteristics.

Further, the Cox regression analysis was conducted to investigate the correlation of clinicopathological factors with BRCA patient OS and DFS based on TCGA BRCA data. Female was set as a reference level for gender, stage 1 for stage, and luminal BRCA for the histological subtype. A *p*-value less than 0.05 was statistically different.

### 2.3 Survival analysis of FN1 methylation

MEXPRESS (https://mexpress.be/) is a data visualization tool designed for the easy visualization of TCGA expression, DNA methylation, and clinical data, as well as the relationships between them. We downloaded the DNA methylation data of FN1 in BRCA for analyzing its prognostic value.

### 2.4 Functional enrichment analysis

The TCGA-BRCA gene expression RNA-seq data were downloaded from the UCSC Xena database (https://xenabrowser.net/) for functional enrichment analysis. The differentially expressed genes (DEGs) according to the median expression of FN1 were screened by setting |log 2 (Fold Change)|>1 and *p* < 0.05 as the thresholds using the limma package. Then, the “ClusterProfiler” package was used to perform Gene Ontology (GO) and Kyoto Encyclopedia of Genes and Genomes (KEGG) analyses of these DEGs. *p* < 0.05 was considered to be statistically significant.

To further elucidate the pathological function of FN1 in BRCA, Gene Set Enrichment Analysis (GSEA) was carried out using the TCGA-BRCA data from the UCSC Xena database. The patients were divided into high and low expression groups by taking the median value of FN1 expression. The gene set was permutated 1,000 times and the expression level of FN1 was used as a phenotypic label. A nominal *p*-value <0.05 and an FDR q-value <0.25 were considered to be statistically significant.

### 2.5 Association of FN1 expression with immune characteristics

To observe the relationship between FN1 expression and tumor immune microenvironment in BRCA, the ESTIMATE algorithm was used to analyze the Immune score and Stromal score. In addition, the TIMER algorithm was employed to determine the association of FN1 mRNA expression with several immune cells including B cell, T cell CD4, T cell CD8, neutrophil, macrophage, and dendritic cell. Finally, Pearson correlation analysis was performed to explore the correlation of FN1 expression with the immune checkpoint gene levels. *p* < 0.05 was considered statistically significant.

### 2.6 Connectivity map analysis

The connectivity map (CMap) can be used to discover the mechanism of action of small molecules, functionally annotate genetic variants of disease genes, and inform clinical trials (Subramanian et al., 2017). The top 300 upregulated and downregulated genes between high- and low- FN1 expression groups were identified as input files of CMap analysis to identify the potential drugs for BRCA.

### 2.7 Statistical analysis

All statistical analyses were performed by SPSS software (SPSS, Inc., Chicago, IL, United States), and R software. The *t*-test was used to analyze differences in each two-group comparison, and one-way ANOVA was employed to assess differences among at least three groups. Survival curves were drawn by the Kaplan-Meier method and differences in survival were compared by logrank tests. All tests were two-sided, and a *p*-value <0.05 was considered statistically significant.

## 3 Results

### 3.1 Patient samples

The FN1 expression data and related clinical information of BRCA patients were retrieved from the cBioportal database based on TCGA. A total of 960 gene expression data profiles and 1,108 clinical data profiles were generated. Patients without complete survival information or gene expression data were excluded. As shown in Table 1, 292 (33.1%) patients aged or under the age of 51 developed BRCA, while 590 (66.9%) patients aged above 51. The majority of patients were females accounting for 98.9%. In this cohort, most patients were at stage 2 (58.4%), followed by stage 3 (23.2), stage 1 (17.0%), and stage 4 (1.4%). Four hundred and seventy-four patients (79.3%) were diagnosed with luminal BRCA, 30 patients (5.0%) as HER2+, and 94 patients (15.7%) as TNBC.

**TABLE 1:** Patient characteristics of breast cancer based on TCGA.

Characteristics	Number of cases	Percentages
Age		
≤51	292	33.1
>51	590	66.9
Gender		
Female	873	98.9
Male	10	1.1
Stage		
Stage 1	147	17.0
Stage 2	506	58.4
Stage 3	201	23.2
Stage 4	12	1.4
Histological subtype		
Luminal	474	79.3
HER2+	30	5.0
Triple-negative	94	15.7

### 3.2 Analysis of FN1 expression

**FIGURE 1:**
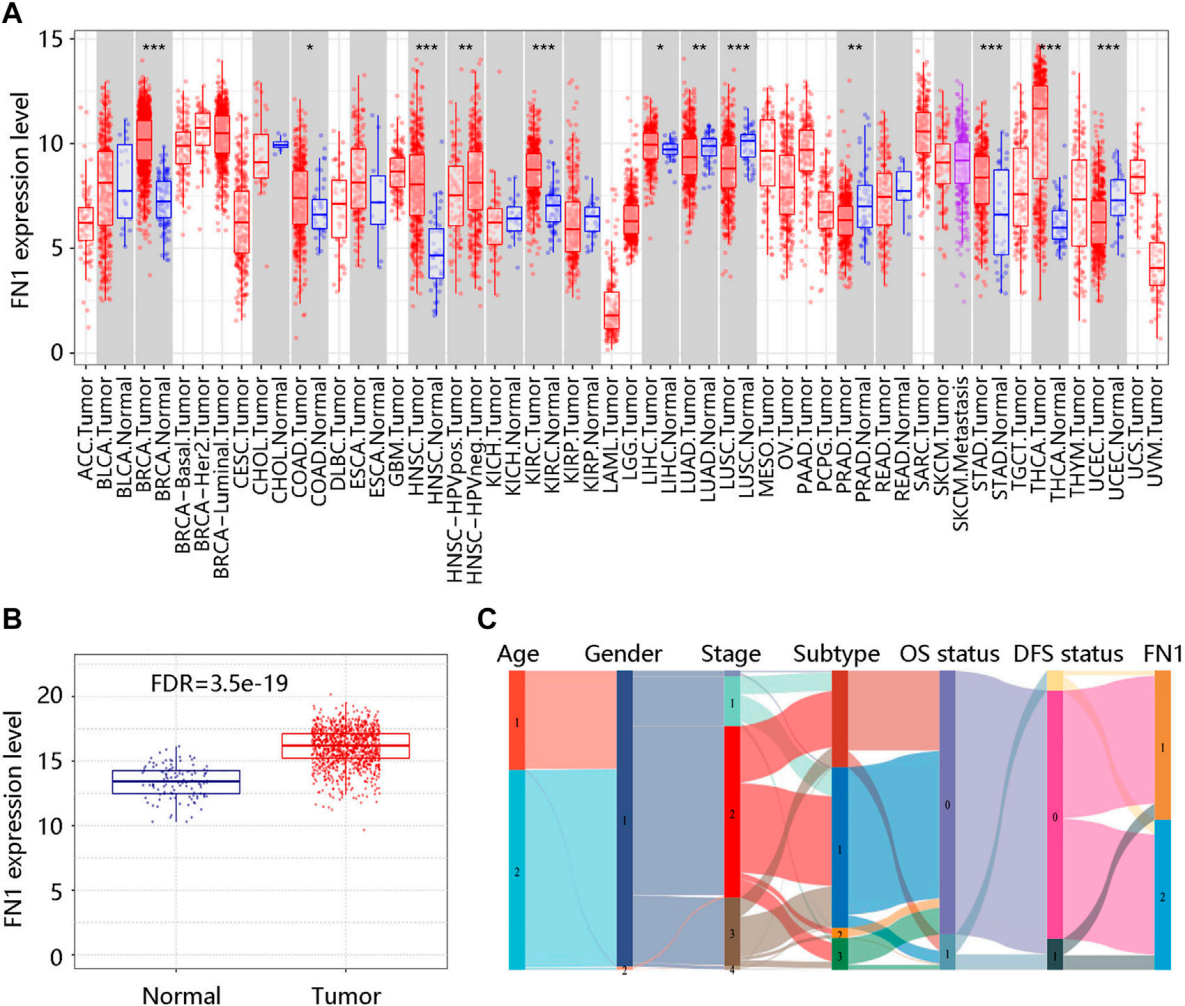
FN1 mRNA expression in breast cancer and other different types of human cancers. **(A)** Significantly different FN1 mRNA expression in 12 tumor types in the TIMER database. *p < 0.05; **p < 0.01; ***p < 0.001. **(B)** Significantly higher FN1 mRNA expression in breast cancer tissue compared with normal breast tissue in the GSCA database. FDR, false discovery rate. **(C)** The association of FN1 expression with different clinical characteristics by the Sankey diagram based on the TCGA BRCA data. Age: 1 represents ≤51 (years), 2 represents >51 (years); Gender: 1 represents female, 2 represents male; Subtype: 1 represents luminal, 2 represents HER2+, 3 represents triple-negative; OS status: 0 represents living; 1 represents deceased; DFS status: 0 represents disease-free; 1 represents recurred; FN1: 1 represents low FN1 mRNA expression, 2 represents high FN1 mRNA expression. OS, overall survival; DFS, disease-free survival.

**FIGURE 2:**
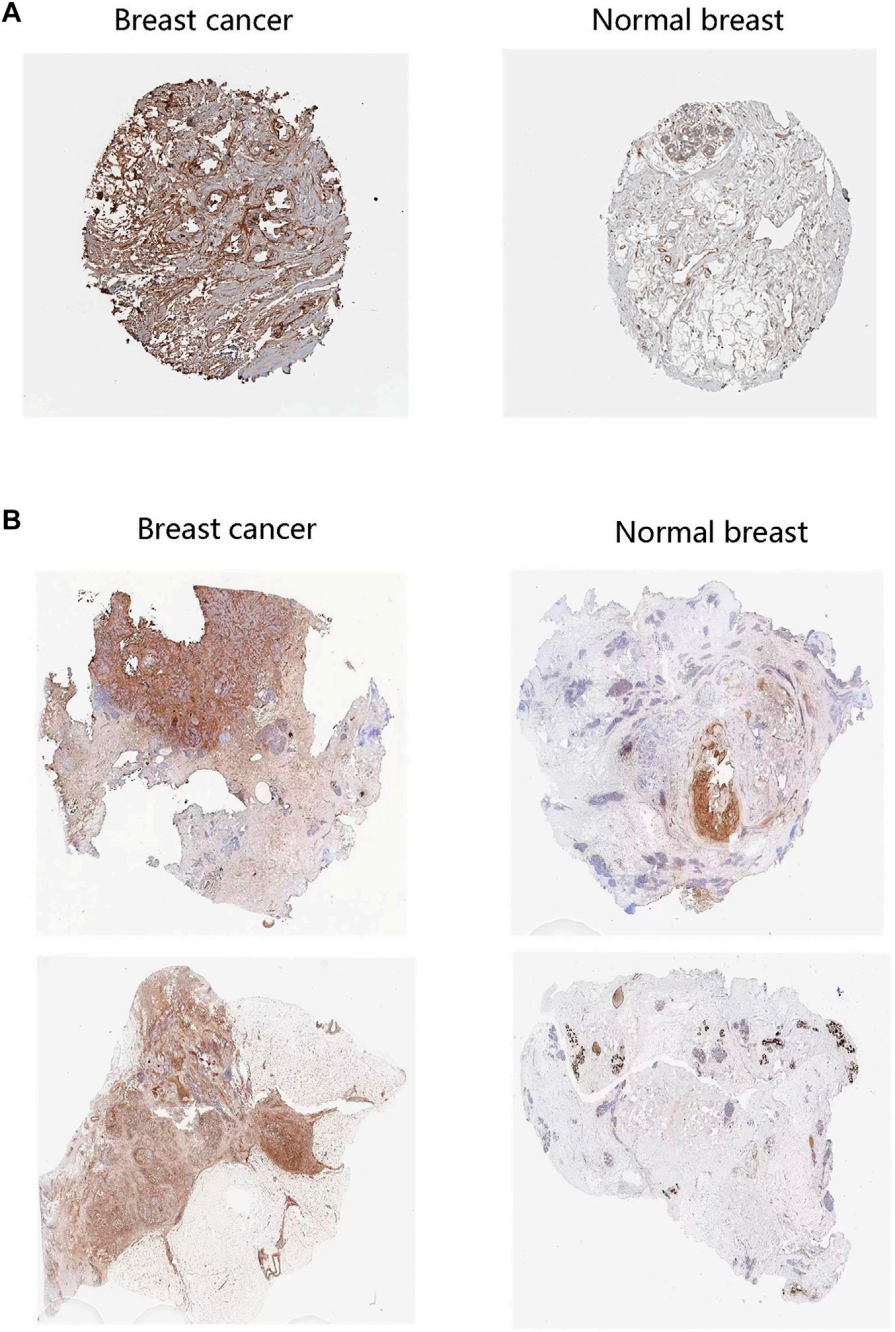
FN1 protein level in breast cancer patients. **(A)** The higher protein levels of FN1 in breast cancer tissues than in normal tissues through the HPA database. **(B)** The higher protein levels of FN1 in breast cancer tissues compared with normal breast tissues by immunohistochemistry.

We firstly analyzed the mRNA expression of FN1 in various tumors as shown in Figure 1A. Analysis of the GSCA database showed that the FN1 mRNA level was obviously increased in BRCA tissues compared with the normal breast tissues (FDR <0.05) (Figure 1B). Sankey diagram was used to exhibit the distribution trend of the high and low FN1 mRNA expression in a different age, gender, stage, histological subtype, OS, and DFS statuses of the BRCA patients (Figure 1C). Besides, the protein level of FN1 was compared in the HPA database. The FN1 protein was highly expressed in the BRCA tissues in comparison to the normal breast tissues (Figure 2A). For validation, we conducted immunohistochemistry to compare the FN1 protein level in BRCA and normal breast tissues. As expected, we observed higher FN1 protein expression in BRCA tissues than that in normal breast tissues (Figure 2B). These results suggested the upregulation of FN1 in BRCA at both mRNA and protein levels.

**FIGURE 3:**
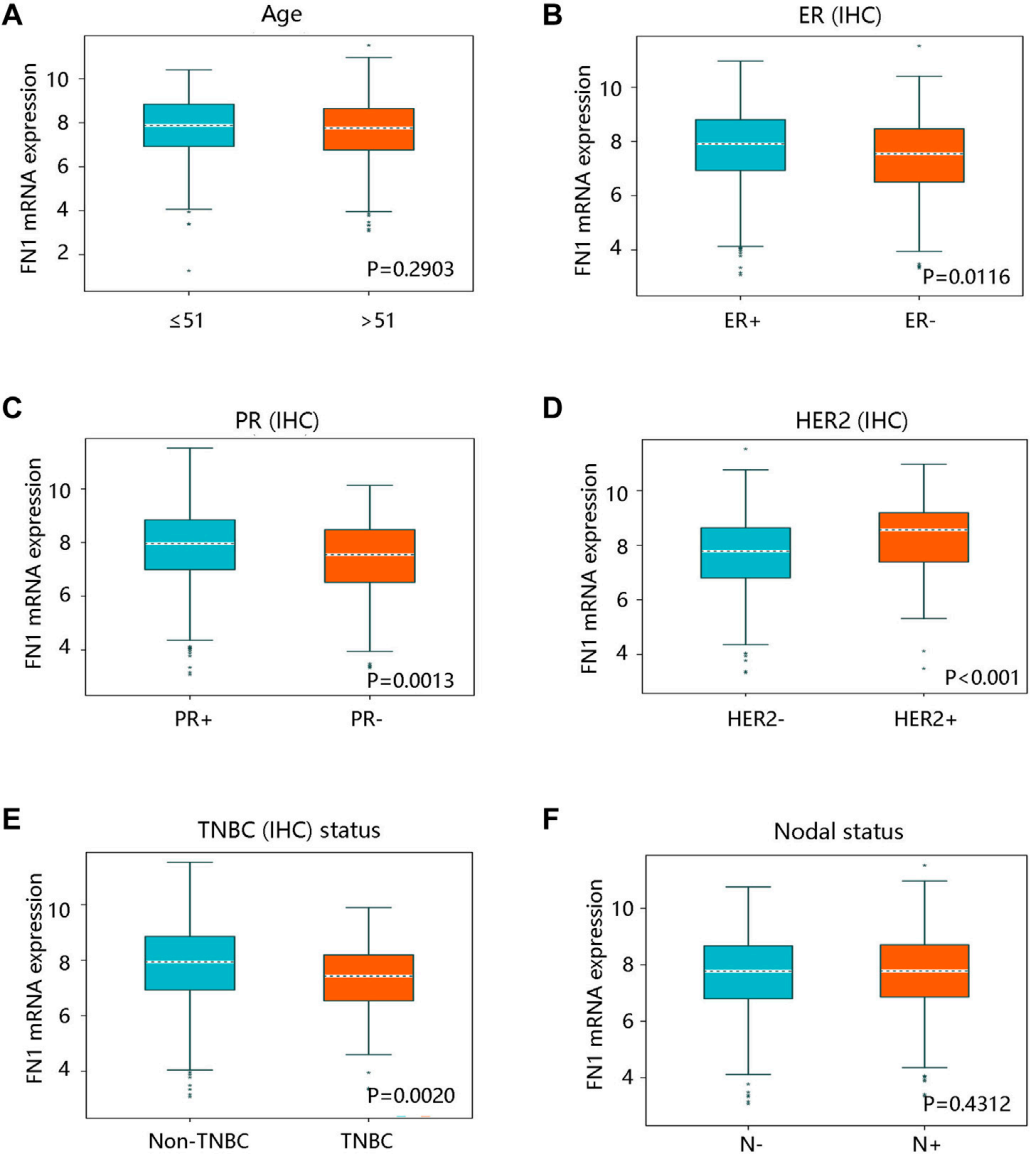
The relationship between FN1 mRNA expression and clinicopathological characteristics in breast cancer in bc-GenExMiner v4.8 database. **(A)** The association of FN1 expression with age with no statistical significance. FN1 expression was significantly related to **(B)** ER status, **(C)** PR status, **(D)** HER2 status, and **(E)** TNBC status. **(F)** No remarkable relationship between FN1 expression and nodal status. IHC; immunohistochemistry; ER, estrogen receptor; PR, progesterone receptor; HER2, human epidermal growth factor receptor 2; TNBC, triple-negative breast cancer.

After characterizing the significant difference in FN1 expression between BRCA and normal breast tissues, we analyzed the clinical factors affecting its mRNA expression by bc-GenExMiner v4.8. There was no statistical difference in FN1 mRNA expression in patients aged no more than 51 vs. over 51 years (*p* > 0.05) (Figure 3A). However, FN1 mRNA expression was apparently decreased in the ER - group vs. in ER + group (*p* < 0.05) (Figure 3B). FN1 mRNA expression was also downregulated in the PR - group vs. the PR + group (*p* < 0.05) (Figure 3C). An elevated mRNA level of FN1 was observed in HER2 + and non-TNBC groups (all *p* < 0.05) (Figures 3D,E). Of note, FN1 mRNA expression was not significantly related to nodal status (*p* > 0.05) (Figure 3F). These findings indicated that the histological subtype might be related to the FN1 mRNA expression.

**FIGURE 4:**
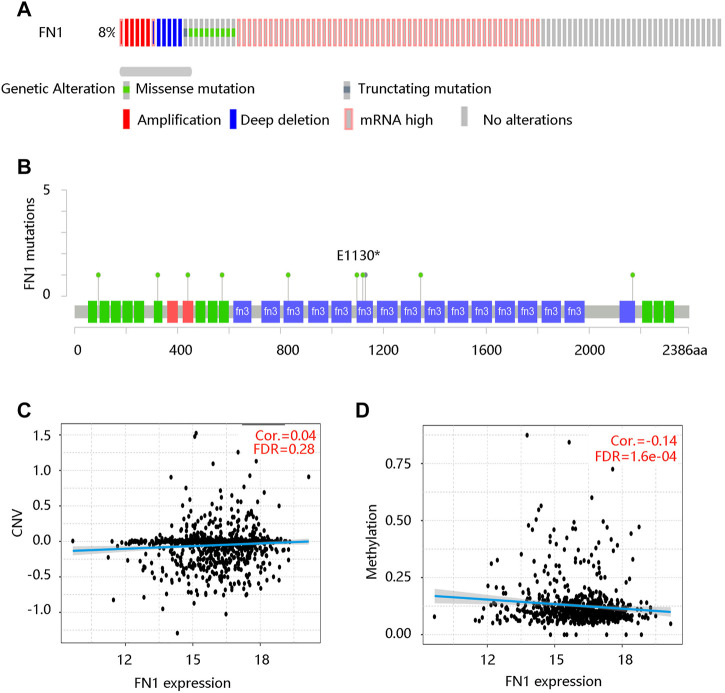
The relationship between FN1 mRNA expression and genetic factors in breast cancer patients. **(A)** The genetic alteration of the FN1 gene. **(B)** The mutated locations of the FN1 gene. **(C)** No significant correlation between FN1 expression and CNV. CNV, copy number variation; FDR, false discovery rate. **(D)** The significantly negative correlation of FN1 expression with its methylation level.

Moreover, we queried the genetic alterations of FN1 in a cohort of 960 BRCA patients (TCGA, Firehose Legacy) on the cBioportal website. The results showed that the FN1 gene was altered in 79 (8%) of queried samples, including missense mutation, truncating mutation, amplification, deep deletion, and mRNA high (Figure 4A). Figure 4B presented the mutated locations of the FN1 gene in the queried BRCA patients. Meanwhile, we analyzed the associations of FN1 mRNA expression with the CNV and methylation levels. FN1 expression was not significantly correlated with the CNV (Figure 4C), but it has a notable negative relationship with its methylation level (Cor. = -0.14, FDR <0.001) (Figure 4D). These results revealed that the genetic alterations and methylation might affect the mRNA level of FN1.

### 3.3 Prognostic value of FN1 expression in BRCA

**FIGURE 5:**
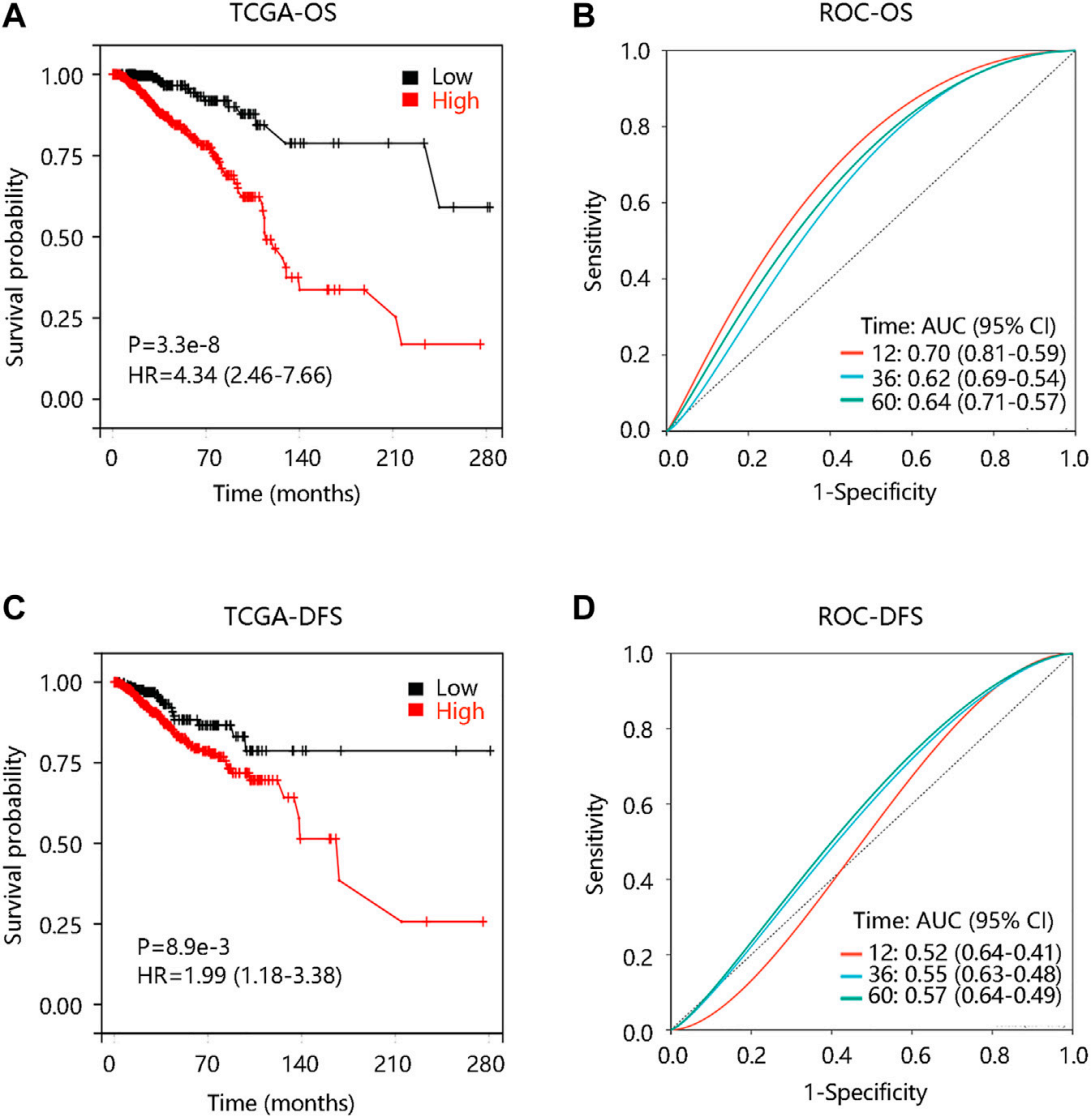
Prognostic analysis of FN1 expression in breast cancer (BRCA) based on TCGA data from the cBioportal database. **(A)** High FN1 expression predicted poor overall survival (OS) in BRCA. **(B)** Receiver operating characteristic (ROC) curve of FN1 expression in predicting OS status in BRCA. **(C)** High FN1 expression predicted poor disease-free survival (DFS) in BRCA. **(D)** ROC curve of FN1 expression in predicting DFS status in BRCA.

**FIGURE 6:**
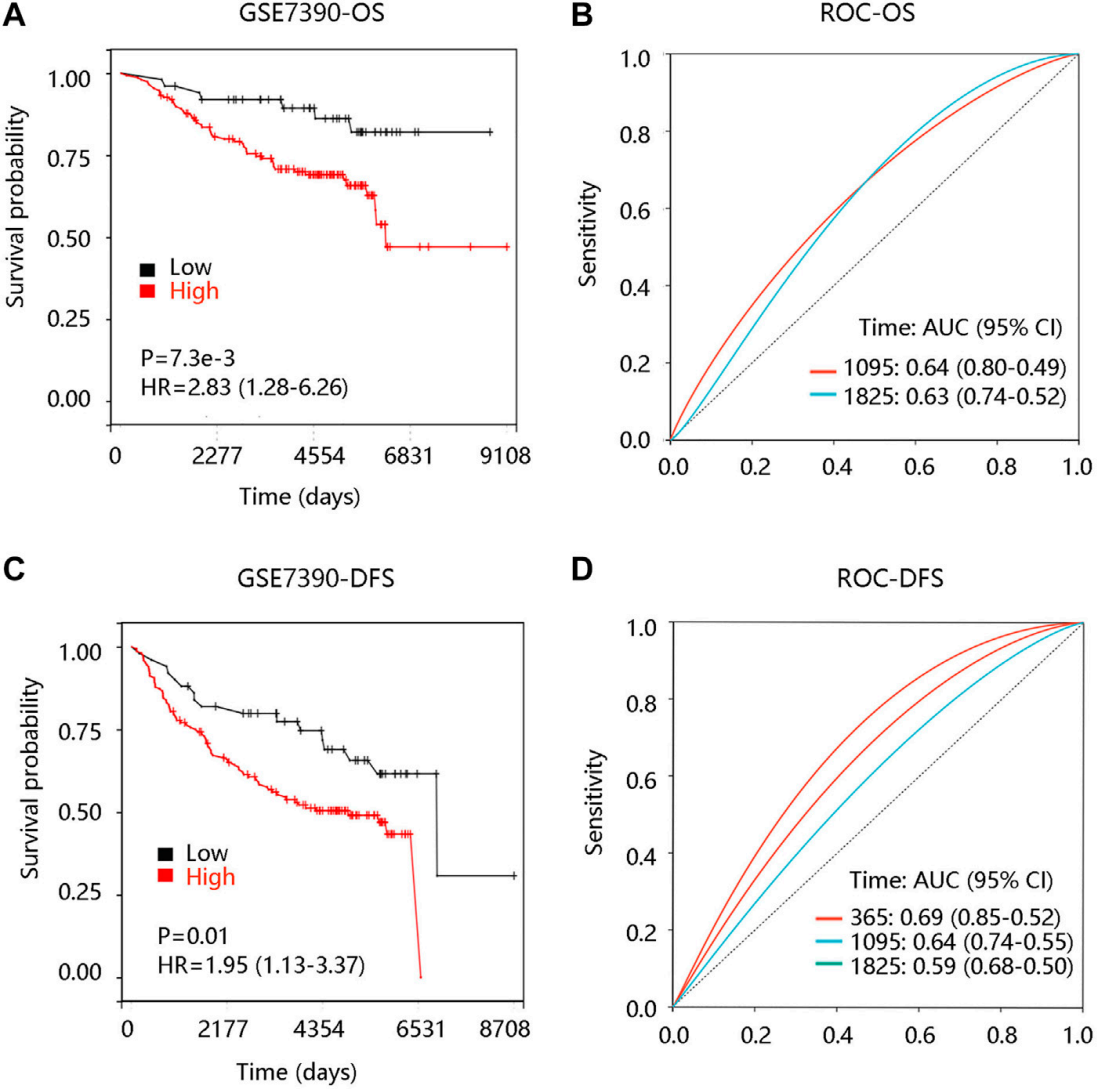
Prognostic analysis of FN1 expression in breast cancer (BRCA) based on the GSE7390 dataset. **(A)** High FN1 expression led to unfavorable overall survival (OS) in BRCA. **(B)** Receiver operating characteristic (ROC) curve of FN1 expression in predicting OS status in BRCA. **(C)** High FN1 expression led to unfavorable disease-free survival (DFS) of FN1 expression in BRCA. **(D)** ROC curve of FN1 expression in predicting DFS status in BRCA.

To investigate the prognostic significance of FN1 expression in BRCA, we evaluated the distribution of FN1 expression and its association with the survival of the patients based on TCGA data from the cBioportal database. The distribution of FN1 mRNA expression and OS status was presented in Supplementary Figure S1A. Specifically, BRCA patients with high FN1 mRNA expression tended to have shorter OS time (*p* < 0.001) (Figure 5A). Besides, the ROC curve was drawn to evaluate the value of FN1 in distinguishing the OS status of BRCA patients by calculating the area under the curve (AUC) (1-year, AUC = 0.70; 3-year, AUC = 0.62; 5-year, AUC = 0.794) (Figure 5B). Meanwhile, we found that FN1 expression had an impact on the DFS status (Supplementary Figure S1B), and higher FN1 mRNA expression contributed to unfavorable DFS time (*p* < 0.01) (Figure 5C). ROC analysis demonstrated that FN1 performed a predictive effect on the DFS status of BRCA patients (AUC = 0.52, 0.55, and 0.57 for 1-year, 3-year, and 5-year, respectively) (Figure 5D). To verify the prognostic and predictive value of FN1 expression in BRCA, the GSE7390 dataset was used for further analysis. Similarly, patients in the high FN1 mRNA expression group possessed worse OS and DFS (all *p* < 0.05), and FN1 had good performance in predicting the OS (3-year, AUC = 0.64; 5-year, AUC = 0.63) and DFS status (1-year, AUC = 0.69; 3-year, AUC = 0.64; 5-year, AUC = 0.59) of BRCA patients (Supplementary Figures S1C, 1D; Figures 6A–D).

Further stratified analysis revealed that a higher expression level of FN1 was significantly correlated with a poor OS and DFS in patients at Grade 2 (*p* < 0.05). FN1 expression had an influence on the OS of TNBC BRCA patients while affecting the DFS of luminal BRCA patients (*p* < 0.05) (Table 2).

**TABLE 2:** The prognostic effect of FN1 expression in breast cancer patients with restricted clinicopathological characteristics.

Characteristics	Overall survival		Disease-free survival	
	HR (95% CI)	*p*-value	HR (95% CI)	*p*-value
Grade				
Grade 1	0.61 (0.24-1.60)	0.32	0.77 (0.44-1.34)	0.35
Grade 2	0.90 (0.60-1.33)	0.59	0.87 (0.70-1.08)	0.21
Grade 3	1.51 (1.11-2.04)	0.0081	1.52 (1.26-1.84)	1.3e-5
Subtype				
Luminal	0.50 (0.23-1.06)	0.064	1.39 (1.04-1.87)	0.026
HER2+	0.51 (0.14-1.80)	0.28	1.79 (0.96-3.34)	0.065
Triple-negative	2.40 (1.26-4.59)	0.0063	1.27 (0.94-1.72)	0.12

Abbreviations: HR, hazard ratio; 95% CI, 95% confidence interval.

To explore the independent prognostic value of FN1 mRNA expression in BRCA, FN1 together with clinicopathological characteristics were integrated into Cox regression analysis. In the univariate analysis, age, stage 2, stage 3, stage 4, TNBC, and FN1 had a close relationship with OS of BRCA patients (all *p* < 0.05) (Table 3). In the multivariate analysis, age, stage 3, stage 4, TNBC, and FN1 were still notably related to OS (all *p* < 0.05) (Table 3). As for DFS, univariate analysis showed that stage 3, stage 4, TNBC, and FN1 were significantly correlated with DFS of BRCA patients, while no significant relationship was found between FN1 and DFS in the multivariate analysis (Table 4). These results indicated that FN1 served as an independent predictor for OS of BRCA patients.

**TABLE 3:** Relationship between clinicopathological characteristics and breast cancer patient OS through Cox regression analysis based on TCGA.

Characteristics	Univariate analysis		Multivariate analysis	
	HR (95% CI)	*p* value	HR (95% CI)	*p* value
Age	1.028 (1.013-1.044)	<0.001	1.050 (1.029-1.071)	<0.001
Gender	1.163 (0.162-8.366)	0.881	0.000 (0.000-Inf)	0.975
Stage 2	2.344 (1.109-4.953)	0.026	1.847 (0.701-4.862)	0.214
Stage 3	4.464 (2.055-9.693)	<0.001	4.600 (1.706-12.402)	0.003
Stage 4	22.347 (7.766-64.306)	<0.001	15.087 (3.952-57.597)	<0.001
HER2+	1.515 (0.464-4.944)	0.491	1.320 (0.401-4.345)	0.648
Triple-negative	1.890 (1.038-3.440)	0.037	2.375 (1.272-4.434)	0.007
FN1	1.950 (1.515-2.509)	<0.001	2.120 (1.432-3.140)	<0.001

Abbreviations: OS, overall survival; HR, hazard ratio; 95% CI, 95% confidence interval.

**TABLE 4:** Relationship between clinicopathological characteristics and breast cancer patient DFS through Cox regression analysis based on TCGA.

Characteristics	Univariate analysis		Multivariate analysis	
	HR (95% CI)	*p* value	HR (95% CI)	*p* value
Age	0.998 (0.981-1.015)	0.780	0.995 (0.971-1.020)	0.714
Gender	0.049 (0.000-339.148)	0.504	0.000 (0.000-Inf)	0.979
Stage 2	1.498 (0.747-3.006)	0.225	1.112 (0.441-2.803)	0.822
Stage 3	3.567 (1.735-7.333)	0.001	2.868 (1.097-7.500)	0.032
Stage 4	13.733 (4.433-42.544)	<0.001	13.019 (3.204-52.894)	<0.001
HER2+	2.752 (0.959-7.898)	0.060	1.836 (0.551-6.121)	0.323
TNBC	2.577 (1.344-4.943)	0.004	2.802 (1.449-5.417)	0.002
FN1	1.324 (1.027-1.709)	0.031	1.297 (0.890-1.890)	0.175

Abbreviations: DFS, disease-free survival; HR, hazard ratio; 95% CI, 95% confidence interval.

### 3.4 Prognostic value of FN1 methylation in BRCA

DNA methylation is an epigenetic alteration that plays an essential role in the development of several cancers (de Almeida et al., 2019). We have unveiled the negative association of FN1 mRNA expression with its methylation levels. Using the DNA methylation data in the TCGA-BRCA cohort, we found that high methylation level groups of cg07533729, cg19773547, cg19727026, cg11309217, cg03228449, cg26950867, cg26910092, cg16261737, cg15127661 contributed to better OS than their related low methylation level groups (all *p* < 0.05) (Table 5).

**TABLE 5:** Summary of the Kaplan-Meier curve data for the nine hyper-methylation sites of FN1 in breast cancer.

Methylation sites	*p*-value	Hazard ratio	Low 95% CI	High 95% CI
cg07533729	1.9e-3	0.52	0.34	0.79
cg19773547	4.1e-3	0.48	0.29	0.80
cg19727026	0.01	0.58	0.38	0.89
cg11309217	7.9e-5	0.44	0.29	0.67
cg03228449	5.1e-3	0.56	0.37	0.84
cg26950867	8.1e-3	0.57	0.38	0.87
cg26910092	0.03	0.64	0.43	0.97
cg16261737	0.01	0.59	0.39	0.91
cg15127661	0.01	0.59	0.39	0.90

Abbreviations: 95% CI, 95% confidence interval.

### 3.5 Functional inference of FN1 in BRCA

**FIGURE 7:**
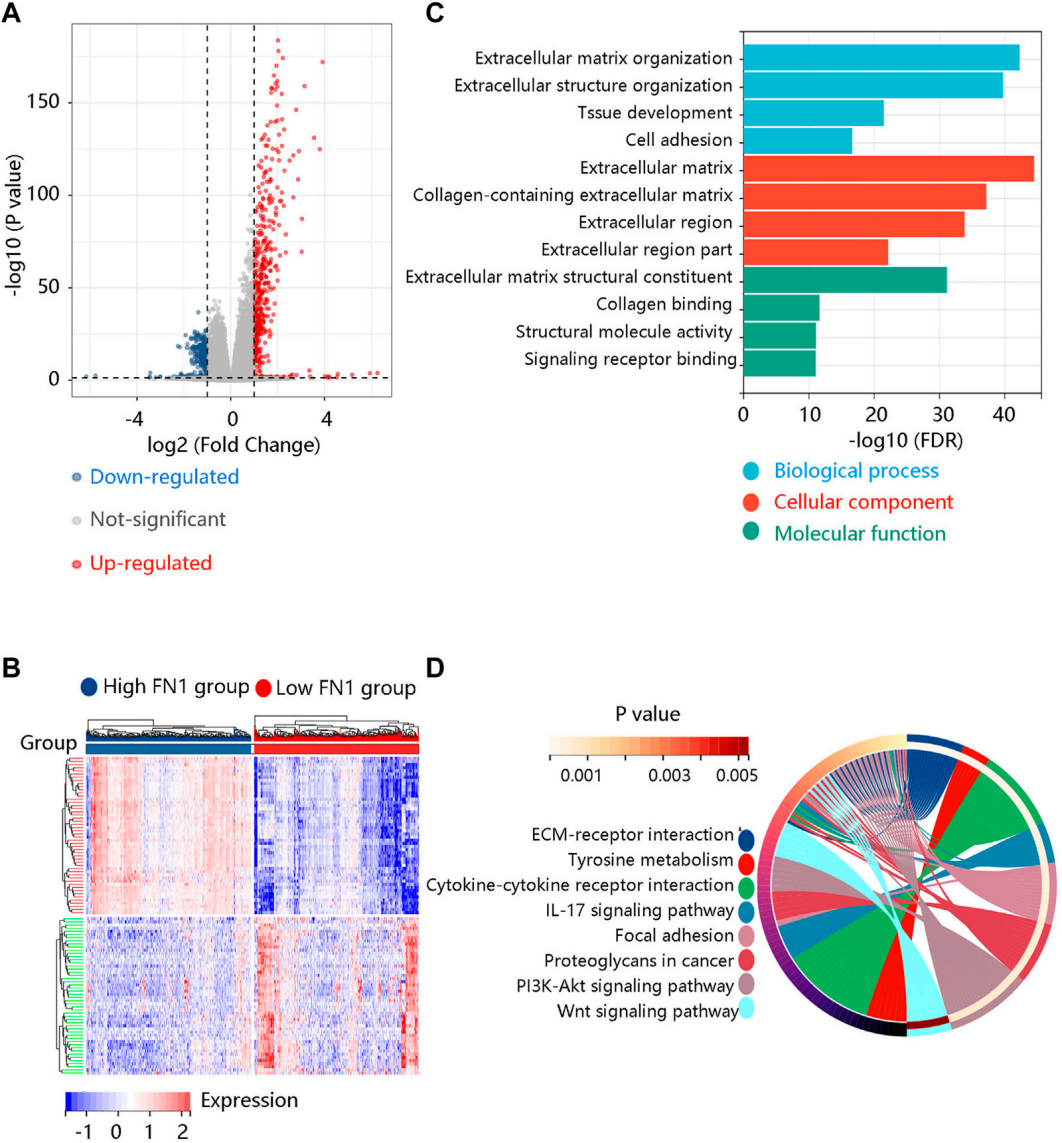
Identification and functional enrichment analysis of the differentially expressed genes (DEGs) between high and low FN1 expression groups. **(A)** The volcano plot of the 480 up-regulated and 398 down-regulated DEGs. **(B)** The heatmap revealed the top 50 DEGs. **(C)** The gene ontology annotations including biological process, cellular component, and molecular function of the DEGs. **(D)** The KEGG pathway enrichment of the DEGs.

DEGs, a popular method to explore the biological role by enrichment analysis, were applied in this study. The volcano plot showed 480 up-regulated and 398 down-regulated DEGs between high and low FN1 expression groups (Figure 7A). Figure 7B displayed the heatmap of the top 50 DEGs. Then, the identified DEGs were loaded for functional enrichment analysis including biological process (BP), cellular component (CC), molecular function (MF), and KEGG pathway analyses. The major BPs were extracellular matrix organization, extracellular structure organization, and cell adhesion; for CCs, they were mainly enriched in the extracellular matrix, extracellular region, and extracellular region part; the primary MFs were collagen binding, structural molecule activity, and signaling receptor binding (Figure 7C). In KEGG analysis, they were involved in ECM-receptor interaction, cytokine-cytokine receptor interaction, focal adhesion, PI3K-Akt pathway, and Wnt signaling pathway (Figure 7D).

**FIGURE 8:**
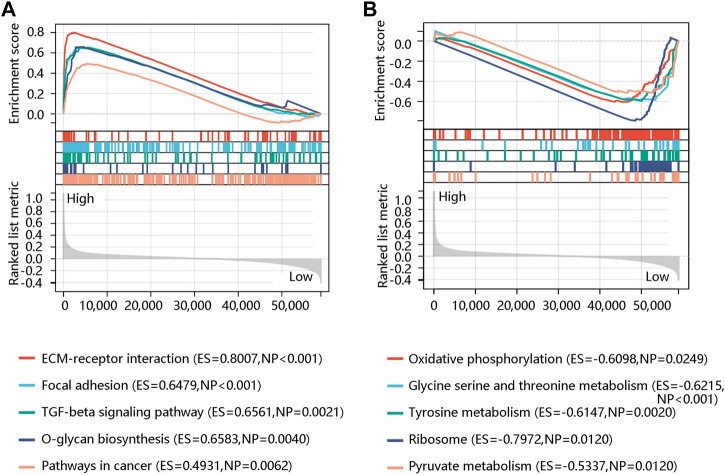
Gene set enrichment analysis showed the top five significant pathways in the high **(A)** and low **(B)** FN1 expression phenotype.

To further reveal the potential pathway that FN1 might regulate the carcinogenesis and development of BRCA, GSEA was performed using the expression data in the TCGA BRCA dataset downloaded from the UCSC Xena database. ECM-receptor interaction, TGF-beta signaling pathway, focal adhesion, O-glycan biosynthesis, and pathways in cancer were significant pathways in the FN1 high-expression phenotype (Figure 8A). Oxidative phosphorylation, glycine serine and threonine metabolism, tyrosine metabolism, ribosome, and pyruvate metabolism were mainly enriched in the FN1 low-expression phenotype (Figure 8B). In summary, FN1 might regulate the occurrence and progression of BRCA through the activation of these pathways, especially ECM-receptor interaction and focal adhesion.

### 3.6 Correlations between FN1 expression and immune characteristics

**FIGURE 9:**
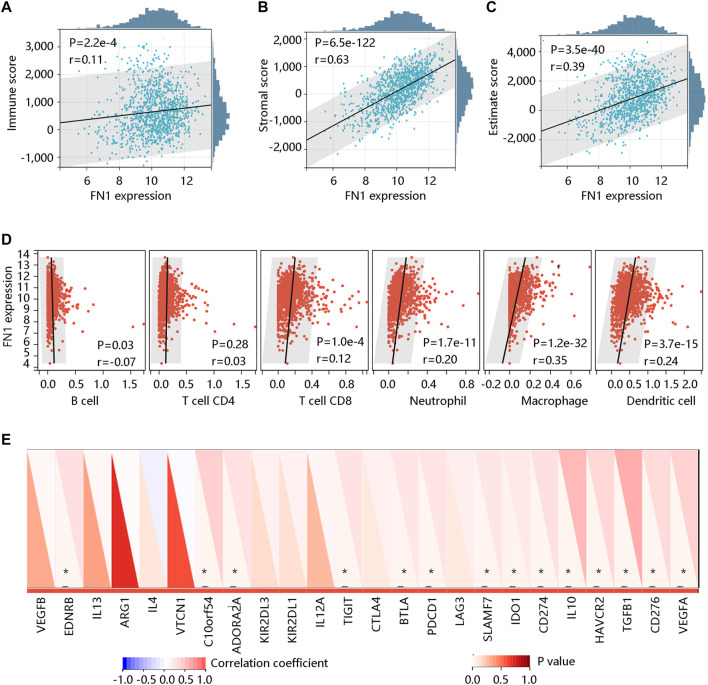
The correlation of FN1 expression with immune cell infiltration in breast cancer. The significantly positive association of FN1 expression with **(A)** Immune score, **(B)** Stromal score, and **(C)** Estimate score using the ESTIMATE algorithm. **(D)** The association of FN1 expression with immune cell infiltration *via* the TIMER algorithm. **(E)** FN1 expression was significantly related to 14 immune checkpoint inhibitors. *p < 0.05.

Tumor-infiltrating lymphocytes are independent predictors for survival in cancer patients (Liu et al., 2021). We determined whether FN1 expression was linked to the degree of immune cell infiltration in BRCA by the ESTIMATE algorithm. The results showed that FN1 expression was positively related to the Immune score (r = 0.11, *p* < 0.001) (Figure 9A), Stromal score (r = 0.63, *p* < 0.001) (Figure 9B), and Estimate score (r = 0.39, *p* < 0.001) (Figure 9C). We next assessed the association of FN1 expression with immune cell infiltration by the TIMER algorithm. Specifically, FN1 was correlated with B cell (r = -0.07, *p* < 0.05), T cell CD8 (r = 0.12, *p* < 0.001), neutrophil (r = 0.20, *p* < 0.001), macrophage (r = 0.35, *p* < 0.001), and dendritic cell (r = 0.24, *p* < 0.001) with statistical differences; whereas, no significant relationship was observed between FN1 expression and T cell CD4 (*p* > 0.05) (Figure 9D). In addition, immune checkpoint inhibitors are a novel strategy for cancer immunotherapy, which improved the clinical outcomes of patients with various cancers (Topalian et al., 2015; Doroshow et al., 2021). Following this, we evaluated the association of FN1 expression with immune checkpoint inhibitors using Pearson correlation analysis. Interestingly, FN1 expression was significantly linked to 14 immune checkpoint inhibitors including EDNRB, C10orf54, ADORA2A, TIGIT, BTLA, PDCD1, SLAMF7, IDO1, CD274, IL10, HAVCR2, TGFB1, CD276, and VEGFA with statistical significance (*p* < 0.05) (Figure 9E). The above findings suggested that FN1 might play an essential role in tumor immunity.

### 3.7 Identification of candidate small molecules

According to CMap analysis result, Telmisartan, Malotilate, seocalcitol, triptorelin, pifithrin-alpha, faropenem, teriflunomide, clomipramine, torcitabine and tiotidine were the top ten potential molecules/drugs for the BRCA patients with high FN1 expression (Table 6).

**TABLE 6:** Identification of top 10 potential therapeutic drugs for breast cancer patients using connectivity map analysis.

Drug	Cell	Dose	*p*-Value
Telmisartan	HCC515	10 uM	0.005
Malotilate	HT29	2.22 uM	0.006
Seocalcitol	U2OS	10 uM	0.009
Triptorelin	MCF7	0.74 uM	0.009
Pifithrin-alpha	A549	70 uM	0.010
Faropenem	THP1	2.22 uM	0.010
Teriflunomide	HA1E	10 uM	0.010
Clomipramine	HA1E	10 uM	0.010
Torcitabine	A375	10 uM	0.010
Tiotidine	U2OS	10 uM	0.0104

## 4 Discussion

BRCA is recognized as one of the most frequent malignancies, which is the leading cause of cancer deaths in women (Bray et al., 2018). Despite the advances in early screenings and medical therapies, the prognosis of BRCA patients remains disappointing because of drug resistance (Chen et al., 2021). Thus, developing the novel biomarkers and exploring the molecular pathogenesis of BRCA for improving the diagnosis and prognosis of this disease is urgently required. As a gene correlated with cell adhesion and ECM remodeling, FN1 inhibits apoptosis, promotes epithelial cell migration, and drives cancer development (Cai et al., 2018). High expression of FN1 was observed in cervical cancer, hepatocellular carcinoma, ovarian cancer, and esophageal cancer (Helleman et al., 2006; Xu et al., 2015; Song et al., 2017; Wang et al., 2019). FN1 overexpression also accelerated the migration and invasion of gastric cancer (Wu et al., 2020). Although FN1 has been demonstrated to exert essential roles in tumorigenesis and tumor progression, the clinical prognostic value and biological function of FN1 have never been illustrated. This is the first systemic research on the expression, prognostic significance, pathological function, and immune-related characteristics of FN1 in BRCA.

In this study, a significant difference was found in the expression of FN1 between BRCA and normal tissues at both mRNA and protein levels. Besides, FN1 was significantly related to ER status, PR status, HER2 status, and TNBC status, but had no significant impact on the age and nodal status. We then analyzed the association of FN1 mRNA expression with genetic factors and found that the FN1 gene was altered in 8% of queried BRCA samples, including missense mutation, truncating mutation, amplification, deep deletion, and mRNA high. Interestingly, FN1 mRNA expression was negatively associated with its DNA methylation level with a statistical difference. DNA methylation patterns were different in the normal and tumor-associated microenvironments, indicating that epigenetic modifications might promote carcinogenesis (Polyak, 2007; Basse and Arock, 2015). Thus, the authors inferred that DNA methylation might regulate the FN1 expression, thereby contributing to the occurrence of BRCA. Another important finding is that the increased expression of FN1 was an independent prognostic factor for predicting worse OS in BRCA patients. Interestingly, the high methylation levels of the FN1 gene at nine methylated sites led to better clinical outcomes.

To reveal the pathological function and underlying mechanism of FN1 in BRCA development, the GO and KEGG analyses of the DEGs between high and low FN1 expression groups were performed. These DEGs were involved in BRCA occurrence and development, such as ECM-receptor interaction, cytokine-cytokine receptor interaction, focal adhesion, PI3K-Akt pathway, and Wnt signaling pathway. Focal adhesion controls cell morphology, adhesion, and migration by connecting ECM and intercellular F-actin, which exerts an essential role in cancer invasion and metastasis, and serves as a crucial determinant in cancer resistance to therapy (Matsuyama et al., 2016; Liang et al., 2018). The abnormal activation of the Wnt signaling pathway was critical in epithelial-to-mesenchymal transition (EMT) activation, accelerating tumor growth and metastasis (Song et al., 2018). Additionally, adherent cells can acquire the ability to migrate through EMT, which is a developmental process in which tumor cells can reactivate EMT to increase their aggressiveness (Aiello and Kang, 2019). Cancer stem cells (CSCs) are correlated with tumor initiation, escape, and recurrence. CSCs may originate from progenitor cells or stem cells in normal tissues, which have self-renewal abilities and are resistant to radiotherapy and chemotherapy (Baumann et al., 2008; Smalley et al., 2013; Zhang et al., 2017). Breast CSCs develop from luminal epithelial progenitors, and the signaling pathways such as PI3K, p53, Hedgehog, Notch, HIF, and Wnt participated in the self-renewal, proliferation, and invasion of breast CSCs (Kasper et al., 2009; Molyneux et al., 2010; El Helou et al., 2017; Shukla et al., 2017; Valenti et al., 2017). Further GSEA results also revealed the ECM-receptor interaction pathway and focal adhesion in the FN1 high expression phenotype. Therefore, FN1 overexpression might promote BRCA occurrence and metastasis, and lead to the unfavorable OS of BRCA patients via the activation of these pathways.

Tumor microenvironments including the stromal influences and macrophages are important in the initiation and progression of BRCA (Maffini et al., 2004; Sonnenschein and Soto, 2016). We found a strong association of FN1 mRNA expression with the Stromal score through the ESTIMATE algorithm. TIMER algorithm results showed that FN1 was positively related to T cell CD8, neutrophil, macrophage, and dendritic cells. The high density of T cell CD8 served as a reliable prognostic and predictor factor for BRCA patients (de la Cruz-Merino et al., 2019). As one of the most common tumor-infiltrating immune cells, macrophages have the ability to generate a mutagenic inflammatory microenvironment, facilitating angiogenesis and helping tumor cells to escape immune rejection (Qian and Pollard, 2010; Dumars et al., 2016; Shan et al., 2020). The number of neutrophils showed a remarkable increase in patients with various cancers. The increased neutrophil-to-lymphocyte ratio was considered as an independent prognostic predictor for worse OS in cancer patients (Huang et al., 2017; Liu et al., 2017; Lee et al., 2018). More importantly, regulation of the PDCD1/PD-L1 axis, a major target of immunotherapy, appears to be one of the major determinants of the efficacy with novel immune-modulated monoclonal antibodies (Herbst et al., 2014; Cimino-Mathews et al., 2016). The PD-L1 expression had an intimate relationship with poor prognosis and aggressive tumor characteristics (de la Cruz-Merino et al., 2019). CD276 is expressed in multiple tumor lines, macrophages, and tumor-infiltrating dendritic cells, which can suppress autoimmunity (Lee et al., 2017). Our study also found a positive correlation of FN1 expression with many immune checkpoint inhibitors such as PDCD1 and CD276. These findings revealed that FN1 might be related to immunity, which might provide new insight into the immunotherapy in BRCA. Of course, further experiments and investigations with larger sample sizes are required to verify these results. Another important finding of this study was the identification of the top 10 potential small-molecule drugs that were predicted based on the DEGs between high- and low-FN1 expression groups in BRCA patients. This provided a novel solution to the unsatisfactory survival of BRCA patients and laid a foundation for further drug research and development.

In summary, bioinformatics analysis as an efficient and accurate method was performed to explore the prognostic and predictive value of FN1 and its association with immune cell infiltrates. FN1 serves as an independent prognostic factor for predicting worse OS in BRCA patients. In addition, FN1 might affect the occurrence and progression of BRCA via regulating immune and inflammation-related pathways.

## Data Availability

The datasets presented in this study can be found in online repositories. The names of the repository/repositories and accession number(s) can be found in the article/Supplementary Material. The link of the original data on Jianguoyun: https://www.jianguoyun.com/p/DQQrfxYQ27KuChj8zrQE.
