# Supplementary figures and images for: FN1 overexpression is correlated with unfavorable prognosis and immune infiltrates in breast cancer
Source: Front Genet. 2022 Aug 12;13:913659. doi: 10.3389/fgene.2022.913659 (PMC9417469; doi:10.3389/fgene.2022.913659)

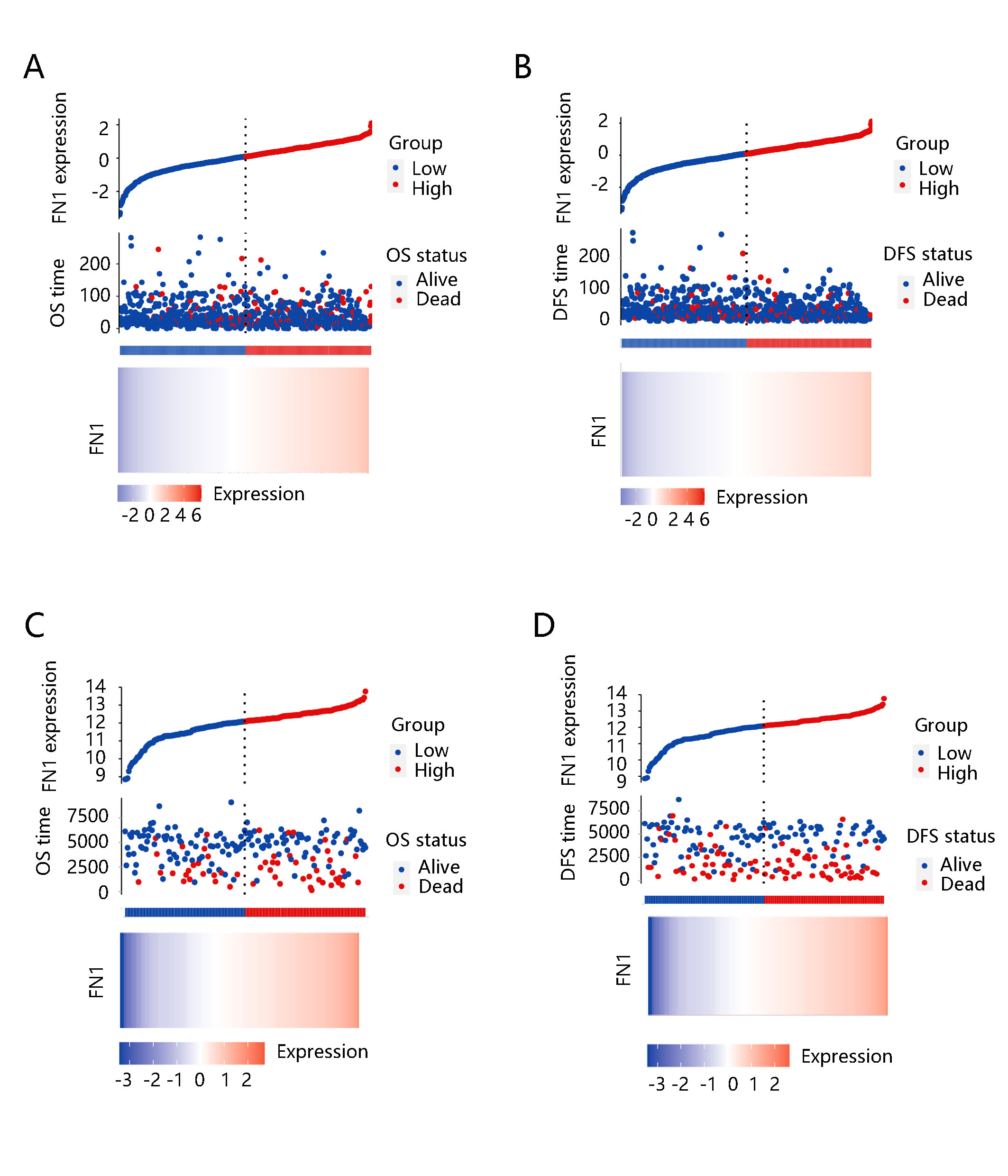

Supplement: Supplementary file 1 [file Image1.jpeg]
